# Supplementary material for: Mechanisms governing the pioneering and redistribution capabilities of the non-classical pioneer PU.1
Source: Nat Commun. 2020 Jan 21;11:402. doi: 10.1038/s41467-019-13960-2 (PMC6972792; doi:10.1038/s41467-019-13960-2)
Supplement: Supplementary file 7 — Source data [file 41467_2019_13960_MOESM7_ESM.zip › Source_Data/FigureS2/FigureS2B_MotifScanOutput/KmeansCluster2/homerResults/motif13.similar.html]

motif13

## Information for motif13

C
G
A
T
T
A
C
G
C
T
G
A
A
C
T
G
A
T
C
G
C
G
T
A
C
T
G
A
G
T
A
C
G
C
A
T
C
T
A
G
A
T
G
C
G
T
A
C
G
T
C
A
  
Reverse Opposite:  

A
C
G
T
A
C
T
G
T
A
C
G
G
A
T
C
C
G
T
A
A
C
T
G
A
G
C
T
A
C
G
T
A
T
G
C
G
T
A
C
G
A
C
T
A
T
G
C
G
C
T
A
  

|  |  |
| --- | --- |
| p-value: | 1e-76 |
| log p-value: | -1.759e+02 |
| Information Content per bp: | 1.707 |
| Number of Target Sequences with motif | 279.0 |
| Percentage of Target Sequences with motif | 5.55% |
| Number of Background Sequences with motif | 493.9 |
| Percentage of Background Sequences with motif | 1.22% |
| Average Position of motif in Targets | 95.1 +/- 53.4bp |
| Average Position of motif in Background | 103.4 +/- 76.0bp |
| Strand Bias (log2 ratio + to - strand density) | -0.4 |
| Multiplicity (# of sites on avg that occur together) | 1.02 |
| Motif File: | file (matrix) reverse opposite |

### Similar de novo motifs found

|  |  |  |  |  |  |  |  |
| --- | --- | --- | --- | --- | --- | --- | --- |
| Rank | Match Score | Redundant Motif | P-value | log P-value | % of Targets | % of Background | Motif file |
| 1 | 0.759 | C T A G C G T A C T A G A C T G C T G A G C T A A T C G C A G T A C T G A G T C A G T C G T C A C T G A | 1e-40 | -94.390295 | 1.43% | 0.10% | motif file (matrix) |
| 2 | 0.752 | G C A T A C T G C T A G A G C T A C G T G A T C G T A C A G C T G T A C G T C A C G T A C T G A | 1e-20 | -47.443668 | 1.08% | 0.16% | motif file (matrix) |
